# Differential repetitive DNA composition in the centromeric region of chromosomes of Amazonian lizard species in the family Teiidae

**DOI:** 10.3897/CompCytogen.v10i2.7081

**Published:** 2016-04-06

**Authors:** Natalia D. M. Carvalho, Edson Carmo, Rogerio O. Neves, Carlos Henrique Schneider, Maria Claudia Gross

**Affiliations:** 1 Laboratório de Citogenômica Animal, Universidade Federal do Amazonas, Instituto de Ciências Biológicas, Estrada do Contorno 3000, Aleixo, CEP 69077-000 - Manaus, AM – Brazil; 2 Laboratório de Tecnologia de DNA, Universidade Federal do Amazonas, Instituto de Ciências Biológicas, Estrada do Contorno 3000, Aleixo, CEP 69077-000 - Manaus, AM – Brazil

**Keywords:** centromere, C*_o_t*1-DNA, FISH, heterochromatin, telomere

## Abstract

Differences in heterochromatin distribution patterns and its composition were observed in Amazonian teiid species. Studies have shown repetitive DNA harbors heterochromatic blocks which are located in centromeric and telomeric regions in *Ameiva ameiva* (Linnaeus, 1758), *Kentropyx calcarata* (Spix, 1825), *Kentropyx pelviceps* (Cope, 1868), and *Tupinambis teguixin* (Linnaeus, 1758). In *Cnemidophorus* sp.1, repetitive DNA has multiple signals along all chromosomes. The aim of this study was to characterize moderately and highly repetitive DNA sequences by C*_o_t*1-DNA from *Ameiva ameiva* and *Cnemidophorus* sp.1 genomes through cloning and DNA sequencing, as well as mapping them chromosomally to better understand its organization and genome dynamics. The results of sequencing of DNA libraries obtained by C*_o_t*1-DNA showed that different microsatellites, transposons, retrotransposons, and some gene families also comprise the fraction of repetitive DNA in the teiid species. FISH using C*_o_t*1-DNA probes isolated from both *Ameiva ameiva* and *Cnemidophorus* sp.1 showed these sequences mainly located in heterochromatic centromeric, and telomeric regions in *Ameiva ameiva*, *Kentropyx calcarata*, *Kentropyx pelviceps*, and *Tupinambis teguixin* chromosomes, indicating they play structural and functional roles in the genome of these species. In *Cnemidophorus* sp.1, C*_o_t*1-DNA probe isolated from *Ameiva ameiva* had multiple interstitial signals on chromosomes, whereas mapping of C*_o_t*1-DNA isolated from the *Ameiva ameiva* and *Cnemidophorus* sp.1 highlighted centromeric regions of some chromosomes. Thus, the data obtained showed that many repetitive DNA classes are part of the genome of *Ameiva ameiva*, *Cnemidophorus* sp.1, *Kentroyx calcarata*, *Kentropyx pelviceps*, and *Tupinambis teguixin*, and these sequences are shared among the analyzed teiid species, but they were not always allocated at the same chromosome position.

## Introduction

Teiidae are a Neotropical lizard family characterized by karyotype diversity with a diploid number ranging from 34 to 52 chromosomes, as well as differences in heterochromatin composition and distribution patterns (Carvalho et al. 2015a, b). Amazonian teiid species exhibit considerable heterochromatic blocks located in centromeric and terminal regions of most chromosomes in *Ameiva ameiva* (Linnaeus, 1758), *Cnemidophorus* sp.1, *Kentropyx calcarata* (Spix, 1825) and *Kentropyx pelviceps* (Cope, 1868), whereas *Tupinambis teguixin* (Linnaeus, 1758) has few heterochromatic blocks in the centromeric regions of macrochromosomes, indicating differential heterochromatin distribution among teiid species (Carvalho et al. 2015a, b).

These heterochromatin blocks usually contain repetitive DNA, such as ribosomal DNA 5S, telomeric sequences, tropomyosin 1 genes, and the retrotransposons *Rex* 1 and *SINE* (Carvalho et al. 2015b). These repetitive elements have been mapped in chromosomes of *Ameiva ameiva*, *Kentropyx calcarata*, *Kentropyx pelviceps*, and *Tupinambis teguixin*. They were mainly located in heterochromatic centromeric and telomeric regions, and appeared to act on the structural organization of the centromere and/or telomere (Carvalho et al. 2015b). However, the composition of the heterochromatic fraction in the genome of *Ameiva ameiva*, *Kentropyx calcarata*, *Kentropyx pelviceps*, and *Tupinambis teguixin* was not restricted to ribosomal DNA 5S sequences, telomeric sequences, tropomyosin 1 gene, and retrotransposons *Rex* 1 and *SINE* because some chromosomes have heterochromatic blocks that are not hybridization signals of these repetitive elements (Carvalho et al. 2015a, b). In *Cnemidophorus* sp.1 the pattern of organization of these sequences is different from other teiids, presenting multiple signals along all chromosomes, with compartmentalized blocks mainly in interstitial chromosome regions (Carvalho et al. 2015b). This indicates differential composition of the centromeric region of this species.

Repetitive DNA may be isolated by various strategies, among them C*_o_t*1-DNA is used to isolate total fraction of moderately and highly repetitive DNA sequences in the genome (Vicari et al. 2010). C*_o_t*1-DNA is based on DNA re-association kinetics, where genome repetitive fractions tend to rapidly reanneal after total genomic DNA denaturation. Thus, average renaturation time of a particular sequence depends on the number of copies found in the genome and in time almost all DNA of a denatured sample will reassociate (Zwick et al. 1997, Ferreira and Martins 2008). Repetitive DNA libraries of various species have identified sequences of microsatellites, satellites, ribosomal DNA, and transposable elements (transposons and retrotransposons) in the genome repetitive fraction (Hřibová et al. 2007, Zhang et al. 2012, Terencio et al. 2015). This DNA library enriched with moderate and highly repetitive sequences (C*_o_t*1-DNA) may be mapped in chromosomes and sequenced, which helps in analysis and has aided in understanding the dynamic and genomic organization, in terms of the repetitive fraction, as well as in evolutionary processes (Ferreira and Martins 2008, Szinay et al. 2010, Yu et al. 2013).

The aim of this study was to characterize sequences of moderately and highly repetitive DNA sequences in *Ameiva ameiva* and *Cnemidophorus* sp.1 genomes. These teiid species have a large amount of heterochromatin that is organized differentially. Libraries enriched with repetitive DNA were cloned, sequenced, used as probes, and chromosomally mapped in *Ameiva ameiva*, *Cnemidophorus* sp.1, *Kentropyx calcarata*, *Kentropyx pelviceps*, and *Tupinambis teguixin*. Furthermore, they assisted in the understanding of genomic sequence organization and dynamics in karyotypes of these Amazonian teiid species.

## Material and methods

Samples of *Ameiva ameiva*, *Cnemidophorus* sp.1, *Kentropyx calcarata*, *Kentropyx pelviceps*, and *Tupinambis teguixin* were collected in Amazonas State, Brazil, in different locations (Table 1). All of the collections were conducted with permission from the Brazilian Environmental Protection Agency (ICMBio/SISBIO 41825-1) (Table 1).

**Table 1.** Species of the Teinae and Tupinambinae subfamilies: collection sites, number and the analyzed animals and voucher specimens (lots) are listed. AM: Amazonas.

Subfamily	Species	Collection sites	Number and sex the analyzed animals	Voucher specimens (lots)
Teiinae	*Ameiva ameiva*	São Sebastião do Uatumã, AM Santa Isabel do Rio Negro, AM Tapauá, AM São Sebastião de Cuieiras, AM Reserva Adolpho Ducke, AM	30 (thirteen males; thirteen females; four without sex identification)	INPA H33213
	*Cnemidophorus* sp.1	Manaus, AM	13 (five males; eight females)	INPA H35018
	*Kentropyx calcarata*	São Sebastião do Uatumã, AM São Sebastião de Cuieiras, AM	7 (three males; four females)	INPA H31712
	*Kentropyx pelviceps*	Tapauá, AM	3 (three females)	INPA H34841
Tupinambinae	*Tupinambis teguixin*	São Sebastião do Uatumã, AM Tapauá, AM Reserva Adolpho Ducke, AM	5 (four females; one without sex identification)	INPA H34791

The animals were euthanized after capture in the field with a lethal dose of the anesthetic sodium thiopental to avoid being deprived of food or water. This research was approved by the Ethics Committee for Animal Experimentation of the Fundação Universidade do Amazonas/Universidade Federal do Amazonas (UFAM) (number 041/2013). No endangered or protected species were used in this research. The animals underwent cytogenetic procedures, were fixed with 10% formaldehyde (injected in the coelom and digestive tract), and preserved in 70% alcohol. Voucher specimens were deposited in the Herpetological Collection of the Instituto Nacional de Pesquisas da Amazônia (INPA H31712, 33213, 34791, 34841, 35018). All samples were identified by the researcher Dr. Federico Arias.

Mitotic chromosomes were obtained from bone marrow cell suspensions *in vitro* using colchicine (Ford and Hamerton 1956). Because the larger heterochromatic regions in *Ameiva ameiva* and *Cnemidophorus* sp.1 represent divergence in the physical chromosomal mapping of different classes of repetitive DNA compared to other teiids, these two species were used to obtain a genomic library enriched with moderately and highly repetitive DNA, following the renaturation kinetics technique C*_o_t*1-DNA (Zwick et al. 1997, Ferreira and Martins 2008). Genomic DNA samples from *Ameiva ameiva* and *Cnemidophorus* sp.1 (50 μl of 100–500 ng/μl of DNA in 0.3 M NaCl) were autoclaved (120°C) for 5 min to obtain fragments between 100 to 2000 bases pairs. Then, samples were denatured at 95°C for 10 min, placed on ice for 10 seconds, and subsequently heated to reannealment at 65°C for 5 minutes. Thereafter, samples were incubated at 37°C for 8 minutes with a unit of S1 nuclease enzyme, whose function is to digest single-stranded DNA. Repetitive fraction of these samples was recovered by freezing in liquid nitrogen and subsequent DNA extraction using phenol-chloroform. The resulting DNA fragments were cloned and sequenced. C*_o_t*1-DNA fragments of *Ameiva ameiva* and *Cnemidophorus* sp.1 were ligated into the plasmid vector pMOSBlue blunt ended (GE Healthcare). Clones were sequenced in an automated ABI 3130 DNA sequencer XL (Applied Biosystem). The alignment of sequences was performed using the Clustal W tool (Thompson et al. 1994) included in the 7.0 BioEdit program (Hall 1999). The generated clones were submitted to BLASTN to detect similarity with public domain sequences contained in the NCBI database (http://www.ncbi.nlm.nih.gov), as well as in the Repbase database (Jurka et al. 2005) from the *Genetic Information Research Institute* (Giri) (http://www.girinst.org/repbase/), using the software CENSOR (Kohany et al. 2006).

C*_o_t*1-DNA libraries were tagged using digoxigenin-11-dUTP for nick translation reaction, according to manufacturer’s instructions (Dig-Nick Translation mix Roche). Anti-digoxigenin rhodamine (Roche) was used for signal detection. C*_o_t*1-DNA libraries from *Ameiva ameiva* tagged using digoxigenin-11-dUTP were hybridized with chromosomes of the species. Further, homologous hybridizations were performed with Cot1-DNA libraries in *Cnemidophorus* sp.1. Probes obtained from C*_o_t*1-DNA of *Ameiva ameiva* and C*_o_t*1-DNA of *Cnemidophorus* sp.1 were also hybridized with chromosomes of other analyzed teiid species. FISH was performed under 77% stringency (2.5 ng/probe, 50% formamide, 10% dextran sulfate, and 2× SSC at 37°C for 18 h) (Pinkel et al. 1986). Chromosomes were counter stained with DAPI (2 mg/ml) in VectaShield mounting medium (Vector).

Chromosomes were analyzed using an epifluorescence microscope (Leica DFC 3000G). Metaphase stages were photographed; the karyotypes were loaded in Adobe Photoshop CS4 software and measured using Image J software. Afterward, the karyotypes were organized following the karyotype formula in karyotypes from *Ameiva ameiva*, *Kentropyx calcarata and Kentropyx pelviceps* were classified as gradual series of acrocentric chromosomes; those of *Cnemidophorus* sp.1 as biarmed, uniarmed, and microchromosomes; and those of *Tupinambis teguixin* as macro and microchromosomes.

## Results

A total of 40 *Ameiva ameiva* Cot1-DNA clones were sequenced wherein 12 sequences corresponded 8 to microsatellites, 1 to transposons, 1 to retrotransposons, and 1 genes, all having high similarity with repetitive DNA deposited in public DNA banks (Table 2). For *Cnemidophorus* sp.1, 30 C*_o_t*1-DNA clones sequenced wherein 8 sequences corresponded 6 to microsatellites, 1 to transposons and 1 genes, all also being highly similar to the repetitive DNAs deposited in public DNA banks (Table 3).

**Table 2.** Repetitive sequences obtained fraction C*_o_t*1-DNA *Ameiva ameiva* with deposited sequences in the NCBI databases and GIRI.

Clone	Homology	Similarity	Identity
AA 1	DNA Transposons	Tc1-like de *Labeo rohita* (GenBank AY083617.1)	100%
AA 2	Microsatellite	Betula platyphylla var. japonica (GenBank AB084484.1)	100%
AA 3	Gene	TAP2 mRNA de *Oryzias latipes* (GenBank AB033382.1)	100%
AA 4	Microsatellite	*Coffea canephora* (GenBank EU526584.1)	100%
AA 5	Microsatellite	*Salmo salar* (GenBank Y11457.1)	96%
AA 6	Microsatellite	*Serranus cabrilla* (GenBank AM049431.1)	95%
AA 7	Microsatellite	*Hypericum perforatum* (GenBank FR732510.1)	93%
AA 8	Microsatellite	*Apteronemobius asahinai* (GenBank AB621739.1)	100%
AA 9	Non-LTR Retrotransposons	CR 1 (RepBase/GIRI*)	88%
AA 10	Microsatellite	*Bos taurus* (GenBank AF271953.1)	81%
AA 11	Microsatellite	*Colias behrii* (GenBank FN552755.1)	100%
AA 12	DNA Transposons	Tc1/mariner (RepBase/GIRI*)	80%

**Table 3.** Repetitive sequences obtained fraction C*_o_t*1-DNA *Cnemidophorus* sp.1 with deposited sequences in the NCBI databases and GIRI.

Clone	Homology	Similarity	Identity
Cn 1	Gene	TAP2 mRNA de *Oryzias latipes* (GenBank AB033382.1)	100%
Cn 2	Microsatellite	Betula platyphylla var. japonica (GenBank AB084484.1)	100%
Cn 3	DNA Transposons	Tc1-like de *Labeo rohita* (GenBank AY083617.1)	100%
Cn 4	Microsatellite	*Colias behrii* (GenBank FN552755.1)	93%
Cn 5	Microsatellite	*Glaucosoma hebraicum* (GeneNank FJ409080.1)	97%
Cn 6	Microsatellite	*Colias behrii* (GenBank FN552755.1)	99%
Cn 7	Microsatellite	*Apteronemobius asahinai* (GenBank AB621739.1)	90%
Cn 8	Microsatellite	*Salmo salar* (GenBank Y11457.1)	96%

**Figure 1.**
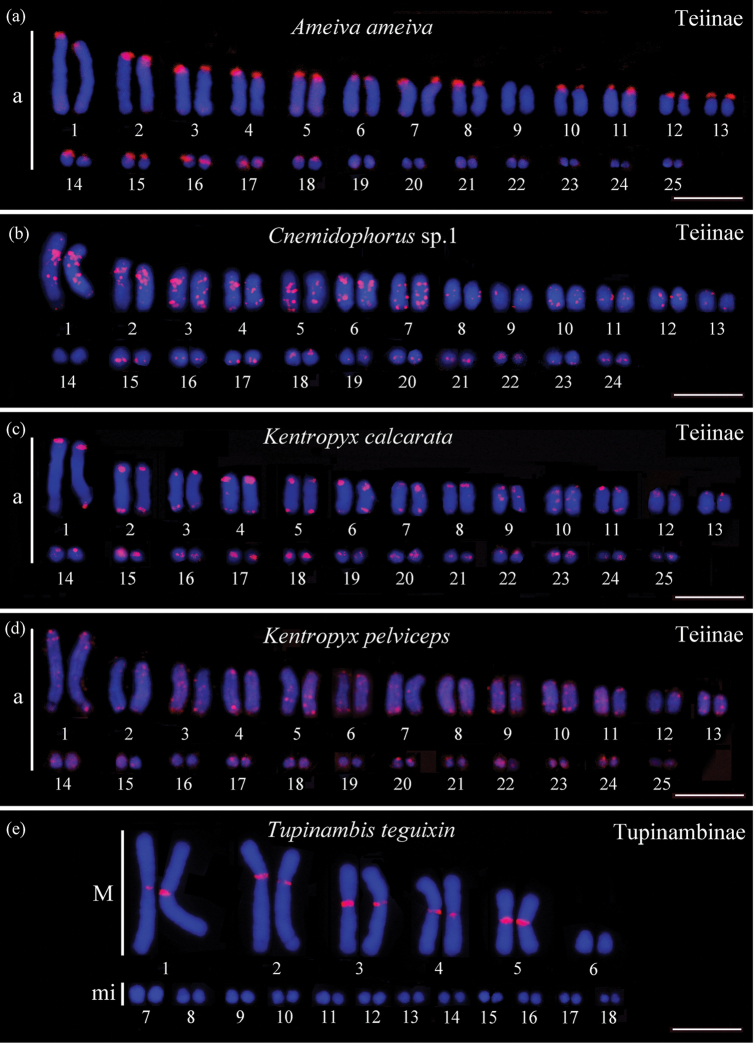
Karyotypes with *Ameiva ameiva* C*_o_t*1-DNA probe hybridized (signal red). **a** *Ameiva ameiva* **b** *Cnemidophorus* sp.1 **c** *Kentropyx calcarata* **d** *Kentropyx pelviceps* and **e** *Tupinambis teguixin*. The chromosomes were counterstained with DAPI. **a** = gradual series of acrocentric chromosomes. **m** = Macrochromosome, **mi** = microchromosome. Scale bar: 10 μm.

By using the homologous probe of C*_o_t*1-DNA, *Ameiva ameiva* hybridization signals were located in the centromeric region/short arm of pairs 1 to 18, except pairs 9, 16, and 17, which showed interstitial signals (Figures 1a). When homologous hybridization of C*_o_t*1-DNA *Cnemidophorus* sp.1 was performed with chromosomes of the species itself, signals were observed in the centromeric region/short arm of certain chromosomes, whereas the majority of chromosomes showed no signals in this chromosome region.

Hybrization using the heterologous probe of C*_o_t*1-DNA obtained from *Ameiva ameiva* in *Cnemidophorus* sp.1 presented multiple signals along all chromosomes, with compartmentalized blocks mainly in interstitial regions (Figure 1b). In *Kentropyx calcarata* and *Kentropyx pelviceps*, signals were located at the centromeric region in the majority of chromosomes, with some pairs having terminal and interstitial signals (Figures 1c and 1d, respectively) and *Tupinambis teguixin* presenting signals in the centromeric region in pairs 1, 2, 3, 4, and 5 (Figures 1e). Similar signal patterns were observed in heterologous hybridization of C*_o_t*1-DNA obtained from *Cnemidophorus* sp.1 in chromosomes of *Ameiva ameiva*, *Kentropyx calcarata*, *Kentropyx pelviceps*, and *Tupinambis teguixin*; however, the signals were more tenuous.

## Discussion

Several classes of repetitive DNA are included in the genome of Amazonian teiid species, such as ribosomal DNA 5S, telomeric sequences, tropomyosin 1 genes, and retrotransposons *Rex* 1 and *SINE*. Most of these repetitive DNA sequences are allocated to heterochromatin regions, in addition to acting structurally in the centromeric and telomeric organization for *Ameiva ameiva*, *Cnemidophorus* sp.1, *Kentropyx calcarata*, *Kentropyx pelviceps*, and *Tupinambis teguixin* (Carvalho et al. 2015b). In addition to these functions, heterochromatin can have other activities in the genome. It can act in chromosome segregation, nuclear organization, mitosis regulation in cell cycle progression, cell proliferation, gene expression regulation, and may affect the process of gene recombination (Grewal and Jia 2007, Skipper 2007, Buhler 2009, Bloom 2014).

However, heterochromatin of teiids is not limited to ribosomal DNA 5S, telomeric sequences, tropomyosin 1 gene, retrotransposons *Rex* 1 and *SINE*, and presents a complex composition with various repetitive DNA. In libraries obtained by C*_o_t*1-DNA sequencing, it was evidenced that different microsatellites, transposons, retrotransposons, some gene families and other type of sequences (e.g. satellite DNAs) are also present in this fraction of moderately and highly repetitive DNA. They were allocated preferentially to the centromeric and telomeric regions of *Ameiva ameiva*, *Kentropyx calcarata*, *Kentropyx pelviceps*, and *Tupinambis teguixin*. These sequences are also present in *Cnemidophorus* sp.1; however, they were present in euchromatic regions, similar to the pattern observed for telomeric sequences, tropomyosin 1 genes, and retrotransposons *Rex* 1 and *SINE*.

The sequences of repetitive DNA that were more abundant in moderately and highly repetitive fractions of the genome obtained using C*_o_t*1-DNA in *Ameiva ameiva* and *Cnemidophorus* sp.1 were microsatellites, which were homologous with sequences of other organisms deposited in public databases, including plants, fish, mammals, bird and insect (Tables 2 and 3). However, the differences in microsatellites isolated from different species are noteworthy. Microsatellite accumulation may be associated with differentiation of sex chromosomes in some species of Sauropsida, because of high suppression of recombination, degeneration, and heterochromatinization (Pokorná et al. 2011, Gamble et al. 2014, Matsubara et al. 2015), but none of the species analyzed in this study had differentiated sex chromosomes; males and females had identical chromosomal constitution.

Microsatellite or simple sequence repeats (SSRs) are short sequences organized in long segments made up of tandem repeat units and are found in coding or non-coding regions in diverse species genomes (de Oliveira et al. 2015). In genetic analysis, they are considered good molecular markers due to its high abundance in the genome, co-dominant inheritance, multi-allelic nature, good reproducibility, and has been used in studies of population genetics, phylogeny, linkage maps, and relationships (Qi et al. 2015, Čížková et al. 2015)

SSRs have functional roles in the genome, such as gene regulation, replication in transcription, protein function, and genome organization (Qi et al. 2015). These SSRs are generally located in centromeric regions and chromosomes ends of various organisms, such as those observed in *Ameiva ameiva*, *Kentropyx calcarata*, *Kentropyx pelviceps*, and *Tupinambis teguixin* in this study, which corroborates the results of other cytogenomic studies involving satellites/microsatellite DNA from some species of lizards (Lacertidae, Scincidae and Varanidae) and snakes (Colubridae, Pythonidae, and Viperidae). These studies showed that the SSRs are located in the heterochromatin region, specifically in the centromeric, pericentromeric, and/or telomeric regions in chromosomes, suggesting differences in the compositions of these regions in the Squamata genome (Singh et al. 1976, Grechko et al. 2005, Chaiprasertsri et al. 2013, Giovannotti et al. 2013, 2014, Matsubara et al. 2015).

In addition to microsatellites, about 50% of sequences obtained by C*_o_t*1-DNA from two species, exhibited similarities to transposable elements (transposons and retrotransposons). One important characteristic of these transposable elements is the transposition mechanism; retrotransposons transpose via an intermediate from RNA and transposons move up the genome through DNA copies that may be contributing to diversity and plasticity of the genome during evolution (Kordis 2009, Kojima et al. 2015). Sequences presented similarity ranging from 80% to 100% with Tc1-like and Tc1/mariner DNA transposons. The Tc1 transposon is found in many eukaryotic genomes and moves into and/or across genomes. It is an element that has defective copies that have been carried across several mutations causing genetic element inactivation in the genome (Ivics and Izsvák 2015, Tellier et al. 2015). Tc1 are not highlighted in the active form in vertebrate genomes (Dornan et al. 2015, Ivics and Izsvák 2015, Tellier et al. 2015).

An retrotransposon, non-LTR retrotransposon CR1 (Chicken Repeat 1) was identified only in the *Ameiva ameiva* genome; however, this does not indicate that it was not present in the genome of *Cnemidophorus* sp.1, because it simply may not have been identified in this study. Retroelement CR1 is a LINE family that is widely distributed in various organisms, including vertebrates (birds, reptiles, and fish) and invertebrates (Thompson et al. 2009). CR1 contains a 5'UTR region, two reading frames (ORFs 1 and 2), and a terminal region 3'UTR. Terminal region 3'UTR have small repeats (microsatellites) relatively conserved within each of the seven CR1 groups (groups A to G) (Suh 2015). The CR1 groups are the only transposable elements that were active during the evolution of birds and reptiles, and thus, have been widely used as phylogenetic markers, in species identification, and for understanding genome evolution within Amniota (Suh et al. 2014, Suh 2015)

Repetitive DNA rDNA 5s, tropomyosin 1 genes, and retroelements *Rex* 1 and *SINE* (Carvalho et al. 2015b), have also been mapped on chromosomes of teiid species analyzed in this study, nevertheless they were not highlighted in sequencing of moderately and highly repetitive DNA obtained by the C*_o_t*1-DNA technique. C*_o_t*1-DNA is product of DNA genomic concentration (C*_o_*), renaturation time in seconds (t), and a constant that depends on buffer cation concentration (Britten and Kohne 1968, Britten et al. 1974). Still, the results of isolated sequences by this technique may be different due to differences in DNA fragmentation (which occur randomly) or cloning processes which may explain failure to obtain repetitive DNAs 5S rDNA tropomyosin 1 genes and retroelements Rex 1 and SINE that also comprise the repetitive fraction of the five analyzed teiid species.

Some clones presented similarity with part of the gene TAP-2 (transporter associated with antigen processing). TAP is encoded by class I major histocompatibility complex (MHC) genes and are responsible for the transport of antigen peptides from the cytoplasm to endoplasmic reticulum (Zhao et al. 2006, Murata et al. 2009). This carrier is comprised of TAP-1 and TAP-2 subunits and is essential in antigen processing and highly conserved among various eukaryotic species (Zhao et al. 2006, Murata et al. 2009). Some genes may be associated with repetitive DNA in different species genomes and may involve various functions, such as genome stability, gene expression regulation, chromatin formation, and as miRNAs (Roberts et al. 2014, Liang et al. 2015). This association of genes with repetitive DNA has been found in some species of lizards, fish, and mammals (Valente et al. 2011, Pokorná et al. 2011, Terencio et al. 2015), and is also evident for analyzed species in this study.

The physical chromosomal map of homologous probes and/or heterologous of C*_o_t*1-DNA showing signals associated with heterochromatic regions in centromeric and telomeric regions in chromosomes of *Ameiva ameiva*, *Kentropyx calcarata*, *Kentropyx pelviceps*, and *Tupinambis teguixin*; and corroborated partially the standard heterochromatic (Carvalho et al. 2015a) the analyzed species. In addition, interstitial signals are also located in interstitial regions of the smaller pairs of chromosomes of *Ameiva ameiva*, *Kentropyx calcarata*, *Kentropyx pelviceps*, and *Tupinambis teguixin*, which confirm the presence of these sequences in this region. However, if we compare the location of these moderately and highly repetitive DNA obtained from C*_o_t*1-DNA with other repetitive DNA already mapped to the same location (Carvalho et al. 2015b), the pattern of signals are similar, being allocated to heterochromatic regions in centromeric and telomeric regions of chromosomes of these teiid species. Although various classes of repetitive DNA can be located in the same chromosomal region from different species, the number of copies of each element may be different (Chaiprasertsri et al. 2013).

In *Cnemidophorus* sp.1, hybridization of moderately and highly repetitive sequences obtained from C*_o_t*1-DNA from *Ameiva ameiva* presented multiple signals along chromosomes with compartmentalized blocks in interstitial regions, which probably are located in euchromatic regions since they are not located in centromeric and telomeric heterochromatic regions of chromosomes (Carvalho et al. 2015a, b). This pattern is similar to the distribution pattern of other repetitive DNA (Carvalho et al. 2015b), indicating that the pattern of chromosomal organization of repetitive elements is different from the other analyzed teiid species. Moreover, repetitive DNA located in euchromatic regions may significantly influence regulatory regions a gene expression because distribution of repetitive elements in the genome, especially the high association to genes with metabolic function, might be the result of a positive selection during evolution and imply practical roles of these elements in gene functions (Wang et al. 2007, Liang et al. 2015). Studies with C*_o_t*1-DNA demonstrated that although mouse and human genomes have similar families of repetitive elements, primary sequences differ significantly among genomes, the human C*_o_t*1-DNA do not hybridize to chromosomes in mice and the same happens with human (Hall et al. 2014). Furthermore, C*_o_t*1-DNA was hybridized on all euchromatic regions of chromosomes (Hall et al. 2014).

On the other hand, FISH using C*_o_t*1-DNA homologous probe revealed signals mainly in centromeric regions of chromosomes of *Cnemidophorus* sp.1. Thus, heterochromatic centromeric fraction of *Cnemidophorus* sp.1 seems to be composed of microsatellites and transposable elements obtained by C*_o_t*1-DNA of the specie itself and elucidated by its sequencing, being different of the sequences obtained by C*_o_t*1-DNA of *Ameiva ameiva*, despite belonging to same categories. Yet C*_o_t*1-DNA contains a variety of different sequences, including the satellite DNA which are among the major component of centromeric heterochromatin, conversely to TE or microsatellite which are interspersed along chromosomes. Other repetitive elements may be present in the heterochromatic fraction of this species, which have not been detected by C*_o_t*1-DNA (Carvalho et al. 2015b). Further, in the centromeric region, other repetitive DNA centromere specifics (Rosic et al. 2014) may be present that may be allocated in both macrochromosomes and microchromosomes, since they share the same repetitive DNA (Giovannotti et al. 2014, Matsubara et al. 2015, this study).

Although its function and multiprotein components are conserved among organisms, these repetitive DNA are extremely divergent regarding its structure, organization, dynamics, and propagation mechanisms, influencing the diversification and evolution of centromeres (Plohl et al. 2014). Therefore, it is clear that several mechanisms may operate in the diversification of these regions, such as unequal crossing-over, gene conversion, changes mediated by transposable elements, and slippage of DNA polymerase replication, resulting in differential composition of heterochromatin in closely related species, among different chromosomes in the same species, or in species-specific chromosomes (Chaiprasertsri et al. 2013, Aldrup-MacDonald and Sullivan 2014, He et al. 2015, Gao et al. 2015).

## Conclusion

This study contributes to understanding the heterochromatic fraction composition and structure and organization of repetitive DNA of teiid genomes and indicates that the different classes of moderately and highly repetitive DNA are part of *Ameiva ameiva*, *Cnemidophorus* sp.1, *Kentropyx calcarata*, *Kentropyx pelviceps*, and *Tupinambis teguixin* genome. This means that these sequences are shared among the analyzed teiid species, although not always allocated on the same chromosome region. Nevertheless, the physical mapping of repetitive DNA revealed similarity among the species *Ameiva ameiva*, *Kentropyx calcarata*, *Kentropyx pelviceps*, and *Tupinambis teguixin* and showed that the centromeric fraction of *Cnemidophorus* sp.1 is different from that of the other analyzed species.

## References

[B1] Aldrup-Macdonald ME Sullivan BA (2014) The past, present, and future of human centromere genomics. Genes 5(1): 33–50. doi: 10.3390/genes5010033 24683489 10.3390/genes5010033 PMC3966626

[B2] Bühler M (2009) RNA turnover and chromatin-dependent gene silencing. Chromosoma 118: 141–51. doi: 10.1007/s00412-008-0195-z 19023586 10.1007/s00412-008-0195-z

[B3] Bloom KS (2014) Centromeric heterochromatin: the primordial segregation machine. Annual Review of Genetics 48: 457–84. doi: 10.1146/annurev-genet-120213-092033 10.1146/annurev-genet-120213-092033 PMC4245377 25251850

[B4] Britten RJ Kohne DE (1968) Repeated sequences in DNA. Science 161(841): 529–540. doi: 10.1126/science.161.3841.529 4874239 10.1126/science.161.3841.529

[B5] Britten RJ Graham DE Neufeld BR (1974) Analysis of repeating DNA sequences by reassociation. Methods in Enzymology 29: 363–418. doi: 10.1016/0076-6879(74)29033-5 4850571 10.1016/0076-6879(74)29033-5

[B6] Carvalho NDM Arias FJ Silva FA Schneider CH Gross MC (2015a) Cytogenetic analyses of five amazon lizard species of the subfamilies Teiinae and Tupinambinae and review of karyotyped diversity the family Teiidae. Comparative Cytogeneics 9(4): 625–644. doi: 10.3897/CompCytogen.v9i4.5371 10.3897/CompCytogen.v9i4.5371 PMC4698576 26753079

[B7] Carvalho NDM Pinheiro VSS Goll LG Carmo EJ Schneider CH Gross MC (2015b) The organization of repetitive DNA in genome organization of amazon lizards species of Teiidae. Cytogenetics and Genome Research 147: 161–168. doi: 10.1159/000443714 26867142 10.1159/000443714

[B8] Chaiprasertsri N Uno Y Peyachoknagul S Prakhongcheep O Baicharoen S Charernsuk S Nishida C Matsuda Y Koga A Srikulnath K (2013) Highly species-specific centromeric repetitive DNA sequences in lizards: molecular cytogenetic characterization of a novel family of satellite DNA sequences isolated from the water monitor lizard (*Varanus salvator macromaculatus*, Platynota). Journal of Heredity 104: 798–806. doi: 10.1093/jhered/est061 24129994 10.1093/jhered/est061

[B9] Čížková J Hřibová E Christelová P Van den Houwe I Häkkinen M Roux N Swennen R Doležel J (2015) Molecular and Cytogenetic Characterization of wild *Musa* Species. PLoS ONE 10(8): . doi: 10.1371/journal.pone.0134096 10.1371/journal.pone.0134096 PMC4529165 26252482

[B10] de Oliveira EA Bertollo LA Yano CF Liehr T Cioffi MB (2015) Comparative cytogenetics in the genus *Hoplias* (Characiformes, Erythrinidae) highlights contrasting karyotype evolution among congeneric species. Molecular Cytogenetics 30: 8–56. doi: 10.1186/s13039-015-0161-4 10.1186/s13039-015-0161-4 PMC4518567 26225139

[B11] Dornan J Grey H Richardson JM (2015) Structural role of the flanking DNA in mariner transposon excision. Nucleic Acids Research 43(4): 2424–2432. doi: 10.1093/nar/gkv096 25662605 10.1093/nar/gkv096 PMC4344528

[B12] Ferreira IA Martins C (2008) Physical chromosome mapping of the Nile tilapia *Oreochromis niloticus* using repetitive DNA sequences. Micron 39: 411–418. doi: 10.1016/j.micron.2007.02.010 17395473 10.1016/j.micron.2007.02.010

[B13] Ford CE Hamerton JL (1956) The chromosomes of man. Nature 178: 1020–1023. doi: 10.1038/1781020a0 13378517 10.1038/1781020a0

[B14] Gamble T Geneva AJ Glor RE Zarkower D (2014) *Anolis* sex chromosomes are derived from a single ancestral pair. Evolution 68(4): 1027–1041. doi: 10.1111/evo.12328 24279795 10.1111/evo.12328 PMC3975651

[B15] Gao D Jiang N Wing RA Jiang J Jackson SA (2015) Transposons play an important role in the evolution and diversification of centromeres among closely related species. Frontiers in Plant Science 7: . doi: 10.3389/fpls.2015.00216 10.3389/fpls.2015.00216 PMC4387472 25904926

[B16] Giovannotti N Nisi Cerioni P Splendiani A Ruggeri P Olmo E Caputo Barucchi V (2013) Slow evolving satellite DNAs: the case of a centromeric satellite in *Chalcides ocellatus* (Forskâl, 1775) (Reptilia, Scincidae). Amphibia-Reptilia 34: 401–411. doi: 10.1163/15685381-00002905

[B17] Giovannotti M Rojo V Nisi Cerioni P González-Tizón A Martínez-Lage A Splendiani A Naveira H Ruggeri P Arribas Ó Olmo E Caputo Barucchi V (2014) Isolation and characterization of two satellite DNAs in some Iberian rock lizards (Squamata, Lacertidae). Journal of experimental zoology Part B Molecular and developmental evolution 322: 13–26. doi: 10.1002/jez.b.22530 10.1002/jez.b.22530 24014193

[B18] Grechko VV Ciobanu DG Darevsky IS Kramerov DA (2005) Satellite DNA of lizards of the genus *Lacerta* s. str. (the group *L. agilis*), the family Lacertidae. Doklady Biochemistry and Biophysics 400: 44–47. doi: 10.1007/s10628-005-0029-3 15846982 10.1007/s10628-005-0029-3

[B19] Grewal SI Jia S (2007) Heterochromatin revisited. Nature Reviews Genetics 8: 35–46. doi: 10.1038/nrg2008 10.1038/nrg2008 17173056

[B20] Hall TA (1999) BioEdit: a user-friendly biological sequence alignment editor and analysis program for Windows 95/96/NT. Nucleic Acids Symposium Series 41: 95–98.

[B21] Hall LL Carone DM Gomez AV Kolpa HJ Byron M Mehta N Fackelmayer FO Lawrence JB (2014) Stable C0T-1 repeat RNA is abundant and is associated with euchromatic interphase chromosomes. Cell 156(5): 907–919. doi: 10.1016/j.cell.2014.01.042 24581492 10.1016/j.cell.2014.01.042 PMC4023122

[B22] He Q Cai Z Hu T Liu H Bao C Mao W Jin W (2015) Repetitive sequence analysis and karyotyping reveals centromere-associated DNA sequences in radish (*Raphanus sativus* L.). BMC Plant Biology 15: . doi: 10.1186/s12870-015-0480-y 10.1186/s12870-015-0480-y PMC4417506 25928652

[B23] Hřibová E Dolezelová M Town CD Macas J Dolezel J (2007) Isolation and characterization of the highly repeated fraction of the banana genome. Cytogenetics and Genome Research 119: 268–274. doi: 10.1159/000112073 18253041 10.1159/000112073

[B24] Ivics Z Izsvák Z (2015) Sleeping beauty transposition. Microbiology Spectrum FAQs 3(2): . doi: 10.1128/microbiolspec.mdna3-0042-2014 10.1128/microbiolspec.MDNA3-0042-2014 26104705

[B25] Jurka J Kapitonov VV Pavlicek A Klonowski P Kohany O Walichiewicz J (2005) Repbase Update, a database of eukaryotic repetitive elements. Cytogenetics and Genome Research 110: 462–467. doi: 10.1159/000084979 16093699 10.1159/000084979

[B26] Kohany O Gentles AJ Hankus L Jurka J (2006) Annotation, submission and screening of repetitive elements in Repbase: RepbaseSubmitter and Censor. BMC Bioinformatics 7: . doi: 10.1186/1471-2105-7-474 10.1186/1471-2105-7-474 PMC1634758 17064419

[B27] Kojima KK (2015) A new class of *SINEs* with snrna gene-derived heads. Genome Biology Evolution (6): 1702–1712. doi: 10.1093/gbe/evv100 26019167 10.1093/gbe/evv100 PMC4494074

[B28] Kordis D (2009) Transposable elements in reptilian and avian (Sauropsida) genomes. Cytogenetic and Genome Research 127: 94–111. doi: 10.1159/000294999 20215725 10.1159/000294999

[B29] Liang KC Tseng JT Tsai SJ Sun HS (2015) Characterization and distribution of repetitive elements in association with genes in the human genome. Computational Biology and Chemistry 57: 29–38. doi: 10.1016/j.compbiolchem.2015.02.007 25748288 10.1016/j.compbiolchem.2015.02.007

[B30] Matsubara K O’Meally D Azad B Georges A Sarre SD Graves JA Matsuda Y Ezaz T (2015) Amplification of microsatellite repeat motifs is associated with the evolutionary differentiation and heterochromatinization of sex chromosomes in Sauropsida. Chromosoma [Epub ahead of print]. 10.1007/s00412-015-0531-z 26194100

[B31] Murata S Yashiroda H Tanaka K (2009) Molecular mechanisms of proteasome assembly. Nature Reviews Molecular Cell Biology 10: 104–115. doi: 10.1038/nrm2630 19165213 10.1038/nrm2630

[B32] Pinkel D Straume T Gray JW (1986) Cytogenetic analysis using quantitative, high sensitivity, fluorescence hybridization. Proceedings of the National Academy of Sciences 83: 2934–2938. doi: 10.1073/pnas.83.9.2934 10.1073/pnas.83.9.2934 PMC323421 3458254

[B33] Plohl M Meštrović N Mravinac B (2014) Centromere identity from the DNA point of view. Chromosoma 123: 313–325. doi: 10.1007/s00412-014-0462-0 24763964 10.1007/s00412-014-0462-0 PMC4107277

[B34] Pokorná M Kratochvíl L Kejnovský E (2011) Microsatellite distribution on sex chromosomes at different stages of heteromorphism and heterochromatinization in two lizard species (Squamata: Eublepharidae: *Coleonyx elegans* and Lacertidae: *Eremias velox*). BMC Genetics 20 12: . doi: 10.1186/1471-2156-12-90 10.1186/1471-2156-12-90 PMC3215666 22013909

[B35] Qi WH Jiang XM Du LM Xiao GS Hu TZ Yue BS Quan QM (2015) Genome-wide survey and analysis of microsatellite sequences in bovid species. PLoS ONE 10(7): . doi: 10.1371/journal.pone.0133667 10.1371/journal.pone.0133667 PMC4510479 26196922

[B36] Roberts JT Cardin SE Borchert GM (2014) Burgeoning evidence indicates that microRNAs were initially formed from transposable element sequences. Mobile Genetic Elements 4: . doi: 10.4161/mge.29255 10.4161/mge.29255 PMC4091103 25054081

[B37] Rošić S Köhler F Erhardt S (2014) Repetitive centromeric satellite RNA is essential for kinetochore formation and cell division. The Journal of Cell Biology 207(3): 335–349. doi: 10.1083/jcb.201404097 25365994 10.1083/jcb.201404097 PMC4226727

[B38] Singh L Purdom IF Jones KW (1976) The chromosomal localization of satellite DNA in *Ptyas mucosus* (Ophidia, Colubridae). Chromosoma 57: 177–184. doi: 10.1007/BF00292916 954552 10.1007/BF00292916

[B39] Skipper M (2007) Genomics: Mysteries of heterochromatic sequences unraveled. Nature 8: 567. doi: 10.1038/nrg2161

[B40] Suh A Churakov G Ramakodi MP Platt RN Jurka J Kojima KK Caballero J Smit AF Vliet KA Hoffmann FG Brosius J Green RE Braun EL Ray DA Schmitz J (2014) Multiple lineages of ancient CR1 retroposons shaped the early genome evolution of amniotes. Genome Biology and Evolution 11(1): 205–217. doi: 10.1093/gbe/evu256 25503085 10.1093/gbe/evu256 PMC4316615

[B41] Suh A (2015) The Specific requirements for CR1 retrotransposition explain the scarcity of retrogenes in birds. Journal of Molecular Evolution 81(1-2): 18–20. doi: 10.1007/s00239-015-9692-x 26223967 10.1007/s00239-015-9692-x

[B42] Szinay D Bai Y Visser R de Jong H (2010) FISH applications for genomics and plant breeding strategies in tomato and other solanaceous crops. Cytogenetic and Genome Research 129: 199–210. doi: 10.1159/000313502 20628252 10.1159/000313502

[B43] Tellier M Claeys Bouuaert C Chalmers R (2015) Mariner and the ITm superfamily of transposons. Microbiology Spectrum 3(2): . doi: 10.1128/microbiolspec.MDNA3-0033-2014 10.1128/microbiolspec.MDNA3-0033-2014 26104691

[B44] Terencio ML Schneider CH Gross MC do Carmo EJ Nogaroto V de Almeida MC Artoni RF Vicari MR Feldberg E (2015) Repetitive sequences: the hidden diversity of heterochromatin in prochilodontid fish. Comparative Cytogenetics 9(4): 465–481. doi: 10.3897/CompCytogen.v9i4.5299 26752156 10.3897/CompCytogen.v9i4.5299 PMC4698564

[B45] Thompson JD Higging DG Gibson TJ (1994) Clustal W: Improving the sensitivity of progressive multiple sequence alignment through sequence weighting, position specific gap penalties and matrix choice. Nucleic Acids Research 22: 4673–4680. doi: 10.1093/nar/22.22.4673 7984417 10.1093/nar/22.22.4673 PMC308517

[B46] Thompson ML Gauna AE Williams ML Ray DA (2009) Multiple chicken repeat 1 lineages in the genomes of oestroid flies. Gene 448(1): 40–45. doi: 10.1016/j.gene.2009.08.010 19716865 10.1016/j.gene.2009.08.010

[B47] Valente GT Mazzuchelli J Ferreira IA Poletto AB Fantinatti BEA Martins C (2011) Cytogenetic mapping of the retroelements *Rex*1, *Rex*3 and *Rex*6 among cichlid fish: New insights on the chromosomal distribution of transposable elements. Cytogenetic and Genome Research 133: 34–42. doi: 10.1159/000322888 21196713 10.1159/000322888

[B48] Vicari MR Nogaroto V Noleto RB Cestari MM Ciofﬁ MB Almeida MC Moreira-Filho O Bertollo LAC Artoni RF (2010) Satellite DNA and chromosomes in Neotropical ﬁshes: methods, applications and perspectives. Journal of Fish Biology 76: 1094–1116. doi: 10.1111/j.1095-8649.2010.02564.x 20409164 10.1111/j.1095-8649.2010.02564.x

[B49] Wang T Zeng J Lowe CB Sellers RG Salama SR Yang M Burgess SM Brachmann RK Haussler D (2007) Species-specific endogenous retroviruses shape the transcriptional network of the human tumor suppressor protein p53. Proceedings of the National Academy of Sciences 703(637): . doi: 10.1073/pnas.0703637104 10.1073/pnas.0703637104 PMC2141825 18003932

[B50] Yu F Lei Y Li Y Dou Q Wang H Chen Z (2013) Cloning and characterization of chromosomal markers in alfalfa (*Medicago sativa* L.). Theoretical and Applied Genetics 126: 1885–1896. doi: 10.1007/s00122-013-2103-z 23636612 10.1007/s00122-013-2103-z

[B51] Zhang L Xu C Yu W (2012) Cloning and characterization of chromosomal markers from a Cot-1 library of peanut (*Arachis hypogaea* L.). Cytogenetic and Genome Research 137: 31–41. doi: 10.1159/000339455 22797674 10.1159/000339455

[B52] Zhao C Tampé R Abele R (2006) TAP and TAP-like—brothers in arms? Naunyn Schmiedebergs Arch Pharmacology 372: 444–450. doi: 10.1007/s00210-005-0028-z 10.1007/s00210-005-0028-z 16525794

[B53] Zwick MS Hanson RE McKnight TD Nurul-Islam-Faridi M Stelly DM (1997) A rapid procedure for the isolation of Cot-1 DNA from plants. Genome 40: 138–142. doi: 10.1139/g97-020 18464813 10.1139/g97-020

